# Cerebral Malformations in Calves Presumed to Be Associated with an Outbreak of Bluetongue Virus Serotype 3 Infection

**DOI:** 10.3390/ani15162359

**Published:** 2025-08-11

**Authors:** Peter Lennart Venjakob, Sarah Schmidt, Patrick Hoch, Daniela Farke, Maximilien Lépine, Kernt Köhler, Walter Grünberg

**Affiliations:** 1 Clinic for Ruminants and Herd Health, Faculty of Veterinary Medicine, Justus-Liebig-University Giessen, 35392 Giessen, Germanywalter.gruenberg@vetmed.uni-giessen.de (W.G.); 2 Small Animal Clinic, Justus-Liebig-University, 35392 Giessen, Germany; 3 Institute of Veterinary Pathology, Justus-Liebig-University, 35392 Giessen, Germany

**Keywords:** arthropod-borne, vector, biting midges, transplacental infection, hydrocephalus internus, hydranencephaly

## Abstract

Bluetongue is a vector-borne viral infectious disease primarily affecting ruminants, transmitted by biting midges of the *Culicoides* species. The first bluetongue virus 3 (BTV-3) cases occurred in Hesse, Germany, in July 2024. From December 2024 onwards, field veterinarians observed calves born with neurological symptoms. A convenience sample of affected calves were admitted to the Veterinary Teaching Hospital at Justus-Liebig-University Giessen, Germany. A total of 13 calves from unvaccinated dams with pronounced neurological symptoms and positive PCR results for BTV-3 were studied. General and neurological examinations were performed and a blood sample was obtained for hematologic, blood biochemical and blood gas analysis. In 11 calves, magnetic resonance imaging (MRI) was performed. Due to the severe neurological lesions, all calves were euthanized and postmortem examinations were performed. The neurological examination of the calves revealed neurological indications consistent with diffuse forebrain disease. MRI revealed that all calves exhibited various stages of cortical parenchyma cell loss and secondary enlargement of the lateral ventricles. Postmortem examination revealed moderate to severe hydrocephalus internus or hydranencephaly. The results of clinical and neurological examinations, MRI, and postmortem examination, indicate severe impairment of brain development presumably associated with transplacental BTV-3 infection. Epidemiologic data and health records from the originating farms suggest that neurologic malformations developed after BTV-3 infection between 95 and 227 of the gestation period.

## Simple Summary

Bluetongue virus is an arthropod-borne infectious disease that affects domestic and wild ruminants. Of the more than 30 serotypes, 24 are pathogenic for ruminants and so far, 1 of them has been shown to cause cerebral malformations in offspring after transplacental infection (bluetongue virus 8). After first being identified in The Netherlands and Germany in 2023, bluetongue virus 3 was first diagnosed in Hesse, Germany, in July 2024. The present case series is a workup of 13 calves born with congenital neurologic symptoms presumably associated with transplacental fetal infection with bluetongue virus 3. Affected calves showed neurological alterations similar to lesions reported earlier in calves born from dams infected with bluetongue virus 8 during pregnancy. The examination reported here, with magnetic resonance imaging and postmortem examination, confirmed similar, severe forebrain lesions, as observed after transplacental infection with bluetongue virus 8.

## 1. Introduction

Bluetongue is a vector-borne viral infectious disease primarily affecting ruminants. The causative bluetongue virus (BTV) pertains to the genus *Orbivirus* of the family of *Reoviridae*. Thus far, over 30 serotypes have been recognized [1,2], of which 24 (1–24) are considered pathogenic for ruminants, and are classified as notifiable to the World Organization of Animal Health [3], and as category C + D + E disease of the European Animal Health Law [4]. Historically, the disease prevailed in the tropic and subtropic regions of the world between the latitudes of 40 to 50° N and 35° S, with peak disease incidences at the seasons of the year with highest vector activity [5]. Over the last few decades, however, outbreaks of BTV have also occurred further north, affecting large parts of central and northern Europe, including the UK [6]. Climate changes with increasing environmental temperatures are thought to contribute to this northward progression of the disease because the presence of the midge vector, population size, biting frequency and survival of the biological vectors required for virus transmission are highly temperature-dependent [6].

Disease transmission occurs through midges of the *Culicoides* species [7]. Only around 30 of the over 1400 recognized *Culicoides* species are susceptible to infection and thus are considered the only competent biological vectors epidemiologically relevant for disease transmission [8].

The primary mechanism of action of the virus is vasculitis and endothelial cell damage, which results in disturbed blood perfusion of affected organs and tissues [9]. Depending on the BTV serotype involved and the ruminant species affected, the clinical presentation of an infection can vary from clinically unapparent to severe systemic and potentially life-threatening disease. Other factors such as breed, age, health, or pregnancy status can further affect the susceptibility of an individual to clinical disease [3].

Until recently, intrauterine BTV infection of fetuses with ensuing malformations, affecting the central nervous system was considered a sporadic incident, primarily observed after inoculation of the dam with a modified live vaccine [10,11]. Following the outbreak of BTV serotype 8 in northern Europe between 2006 and 2008, a number of reports of malformations of the central nervous system in calves were published [12,13,14]. In the present case series, we report the diagnostic workup of 13 calves with congenital neurologic abnormalities born between November 2024 and February 2025, following an outbreak of BTV serotype 3 (BTV-3) that took place in Germany starting in Spring 2024.

## 2. Field Situation

In September 2023, BTV-3 was identified for the first time on the European continent in four sheep flocks in the Dutch province of Noord Holland [15]. From there, the infectious disease spread eastward, with the first cases reported in Germany in October 2023 in North Rhine Westphalia and Lower Saxony. With BTV being a notifiable disease, animal transport restrictions were enforced immediately in the affected regions. In July 2024, the first BTV-3 cases occurred in Hesse, Germany, with peak case numbers recorded in August and September of that year [16]. Inactivated vaccines against BTV-3 authorized for the use in ruminants per emergency decree were available from June 2024 in the European Union, but vaccination was not widely used until the end of the year [17]. As expected, based on the nature of this vector borne disease, the number of recorded cases decreased considerably between September and November 2024 [16].

From November 2024, the Cattle Health Service in Hesse, Germany, received initial reports from field veterinarians describing an increased number of calves born with neurologic symptoms such as central blindness, insufficient suckling behavior, intermittent seizures, or stereotypic behavior such as compulsive tongue playing or head pressing. These cases were not immediately linked to the BTV-3 epidemic that affected large parts of Germany, and specifically Hesse, a few months earlier. Veterinarians and producers reported unsuccessful treatment attempts with antimicrobials, steroidal and non-steroidal anti-inflammatory drugs, or injectable vitamins. Initially, reports primarily concerned cow–calf operations, with calving seasons starting in November. Later, similar cases were also reported in dairy farms. The number of observed cases peaked during the holiday season between Christmas 2024 and New Year 2025, with some veterinarians reporting several cases on different farms on the same day. Because of the similarities of the reports with cases observed during an outbreak of BTV serotype 8 (BTV-8) in Europe in 2006/2007, the blood of some of the affected calves and their dams was analyzed for BTV-3 antigen and antibody. Several of the analyses yielded a positive result for BTV-3 antigen in affected calves and PCR-negative and BTV antibody-positive results in unvaccinated dams. A convenience sample of calves with laboratory results as mentioned above were admitted for diagnostic workup to the veterinary teaching hospital (VTH) of the Justus-Liebig-University Giessen in Germany.

## 3. Case Selection and Diagnostic Workup

Only calves from unvaccinated herds, with a positive result for BTV-3 antigen, born from dams with a BTV antibody titer were included in the present case series. Written consent to include each animal in the present case series was obtained from all owners.

At admission, calves underwent a general physical exam and a detailed neurologic exam. The physical exam was performed following established standard procedures [18]. A venous catheter (VasoVet venous catheter, 14G—2.2 × 50 mm, Braun, Melsungen, Germany) was placed and secured in one of the jugular veins. Blood was collected anaerobically in lithium-heparin and EDTA tubes for further analysis. Heparinized whole blood was used for blood gas analysis that was conducted within 30 min of sampling. The remaining heparinized blood and EDTA blood was used for further biochemical and hematologic examination. An EDTA blood sample was also submitted to the Federal Laboratory of Hesse, Germany, (Landesbetrieb Hessisches Landeslabor, Giessen, Germany) for the detection of BTV-3 antigen and BTV antibody. A PCR approved by the national reference laboratory at the German Friedrich-Loeffler-Institute was used for the detection of BTV and BTV-3 antigen [19]. Furthermore, all calves were tested for the presence of Bovine Viral Diarrhea virus (BVD) by ear notch examination, an imperative analysis conducted in all calves at the time of birth as part of the German BVD eradication program [20]. In order to be eligible to leave the farm of origin, the BVD test is required to be negative. Calves were also tested for the foot and mouth disease antigen, with negative results.

Calves were then scheduled for a magnetic resonance imaging (MRI) exam under general anesthesia. General anesthesia was induced by 0.07 mg/kg xylazin and 4 mg/kg ketamine, followed by endotracheal intubation and maintaining anesthesia with 1.2% isoflurane and ketamine drip with a constant infusion rate of 0.2 mg/kg/h. Imaging of the head was then performed with a 1.5 Tesla high-field MRI scanner (Siemens Verio, Siemens Healthcare, Erlangen Germany). Images included at least sagittal, transverse, and dorsal T2-weighted images (Turbo Spin Echo, Siemens Healthineers, Erlangen, Germany, TR 6280 ms, TE 123 ms, slice thickness 2.5 mm), transversal FLAIR weighted images (TR 9000 ms, TE 99 ms, slice thickness 3 mm) and 3D-T1-weighted pre- and post-contrast medium administered images (TR 13 ms, 5 ms, and slice thickness 0.7 mm). After completion of the MRI, all study animals were euthanized with 0.3 mL/kg pentobarbital while still under general anesthesia due to severe neurological symptoms, in combination with the results of the MRI study, and the ensuing poor prognosis, and were then submitted for immediate postmortem examination. In two instances, calves did not undergo an MRI exam due to poor general health. One calf was emaciated and unable to drink. The other calf presented with a subluxation of the left glenohumeral joint. These two calves were humanly euthanized shortly after physical examination and blood sampling and were directly submitted for postmortem examination. For each case, an attempt was made to crudely estimate the stage of fetal development at the time of infection of the dam with BTV-3. In cases where clinical disease associated with BTV infection was observed either in the pregnant dam or in the herd, this period was considered as the most probable time of infection of the dam. In several cow–calf operations with the herd on pasture during the summer months, clinical disease was not observed in the herd. In these cases, the date (±15 d) where first cases of BTV-infections were recorded in the specific area of the operation in the national Animal Disease Information System [16] were considered as most likely time of infection. The age of the fetus at the time of infection was calculated either from the date of artificial insemination in the case of dairy calves, or was calculated from the date of birth assuming an average duration of pregnancy of 282 days in calves born in cow–calf operations.

## 4. Diagnostic Results

### 4.1. Study Population

A total of 13 calves with neurologic abnormalities present since birth were eligible for inclusion in the present case series. The neurological abnormalities described by the animal owners or referring veterinarians included tremors (n = 1), disorientation (n = 3), opisthotonus (sometimes referred to as “stargazing”; n = 4), aimless chewing (n = 2), and central blindness (n = 1). Other symptoms mentioned in the history included skin lesions on the muzzle (n = 1) and reduced suckling behavior (n = 5). The animals that were referred by veterinarians of the greater area surrounding the VTH originated from farms in Hesse, eastern North Rhine–Westphalia, Rhineland–Palatinate and northern Bavaria, and were all born between November 2024 and February 2025. Calves enrolled in the present case series originated from dairy farms (n = 6) and cow–calf operations (n = 7). All dairy calves were Holstein Friesians. The calves originating from cow–calf operations were Simmental, Aberdeen Angus (n = 2), Charolais, Limousin, Gelbvieh, and Rotvieh calves. The age at admission ranged from 3 to 70 days. The following treatments were administered by the owner or referring veterinarian: antimicrobials (n = 2), non-steroidal anti-inflammatory drugs (n = 3), vitamin supplements (n = 3), parenteral iron application (n = 1), intravenous glucose infusions (n = 1), and steroids (n = 1). Since none of the calves responded to the treatment, the calves were referred to the VTH with the understanding that animals would be humanely euthanized and primarily used for in-depth diagnostic workup.

### 4.2. Genome Analysis for Infectious Diseases

The analysis used to identify BTV antigen in samples obtained at admission confirmed a positive result in all 13 cases. In 10 of these cases, a specific BTV-3 PCR also yielded positive results. In one case, the latter analysis was negative, and in two cases the specific BTV-3 PCR was not conducted because this serotype was previously identified on the farm.

The earnotch analysis for BVD yielded a negative result in all cases. Similarly, the foot and mouth disease virus PCR yielded negative results in all cases.

### 4.3. Physical Examination

**Figure 1:**
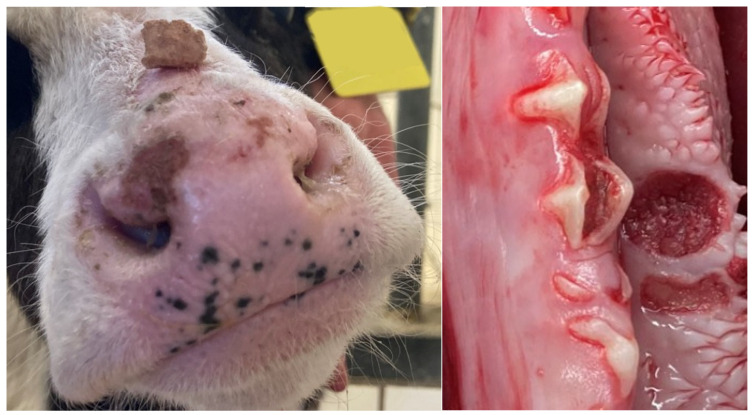
Lesions on the muzzle and local mucosal erosion (left mandible in the area of the 2nd molar tooth) in calves after intrauterine infection with bluetongue virus serotype 3.

At admission, all studied calves were able to rise, stand, and walk without assistance, with two exceptions. One of the two recumbent calves showed profuse diarrhea with marked dehydration, hypoglycemia, and hypothermia, while the second calf was diagnosed with an apparently traumatic subluxation of the left glenohumeral joint. The remaining calves showed varying degrees of ataxia. Overt profuse diarrhea was diagnosed in four calves. In four cases, animals were diagnosed with moderately decreased body condition. Subnormal rectal temperature was found in two cases (34.4 and 35.5 °C), while five calves had elevated rectal temperature (39.7 to 42.3 °C). Dehydration between 5 and 8%, as estimated from eyeball recession [21], was diagnosed in three instances. Tachycardia (heart rate over 120 beats per minute) with frequencies between 124 and 152 was diagnosed in four instances. Lung auscultation revealed abnormal inspiratory breathing sounds in nine calves, and tachypnoea in six calves. Inspection of the muzzle and oral mucous membranes revealed local erosions in four animals (Figure 1). The results of the physical examination, imaging and postmortem examination are summarized in Table 1.

### 4.4. Hematology and Blood Biochemstry

The results of the hematologic exam, standard plasma biochemistry, and blood gas analysis are summarized in Supplementary Table S1. The leukogram revealed moderate leukocytosis with concomitant granulocytosis in two calves, and one animal with mild leuko- and lymphopenia. Five of the calves had platelet counts above the reference (>1022 × 10^9^/L).

Elevated packed cell volumes were determined in two calves, while the red blood cell counts were within the reference range for calves (5.4–10.6 *×* 10^12^/L) in all instances. The blood hemoglobin concentrations were below the reference range of 4.7 mmol/L in two instances, with one of these calves also showing subnormal mean corpuscular volume (MCV).

Plasma biochemistry revealed plasma protein concentrations below the reference range (59.2–87.5 g/L) for calves in nine animals. Hypoproteinemia was attributable to subnormal albumin and globulin concentrations in four calves, attributable to hypoglobulinemia only in three calves, and attributable to hypoalbuminemia only in two calves. Five calves had mildly to moderately increased blood urea nitrogen concentrations. In one beef calf (28 d old Aberdeen Angus weighing 53 kg at admission), an increased creatinine concentration of 159 µmol/L was measured. Four calves had increased concentrations of total bilirubin. Elevated glutamate dehydrogenase (GLDH) activities were determined in six calves, with values above 100 IU/L (reference < 30 IU/L) measured in two instances.

The blood gas analyses revealed mild acidemia in seven calves that was associated with mildly decreased base excess in six cases, and with elevated L-lactate concentrations in one instance. None of the calves with subnormal blood pH showed pCO_2_ concentrations outside the reference range, thus ruling out a respiratory cause of the negligible metabolic acidemias. In four cases, acidemic animals showed slightly decreased strong ion difference (SID), with values between 36 and 39 mEq/L, suggesting a mild strong ion acidosis [22].

### 4.5. Neurologic Examination

All examined calves showed neurological symptoms consistent with diffuse forebrain disease. Specifically, calves were presented with obtundation (n = 13), absent menace response and preserved pupillary light reflex (n = 13), ventrolateral strabismus (n = 13), proprioceptive deficits (n = 13), ambulatory tetraparesis with proprioceptive ataxia of all four limbs (n = 9), opisthotonus (n = 2), compulsive movements (n = 2), and compulsive tongue playing (n = 1). One calf presented with horizontal nystagmus.

### 4.6. Imaging

**Figure 2:**
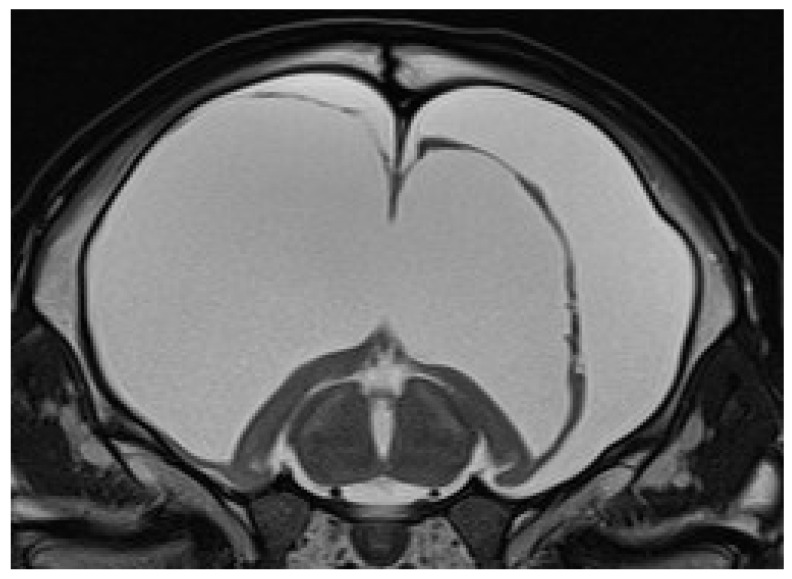
Transverse T2-weighted MRI of a brain at the level of the thalamus and hippocampus of a 24-day-old male calf with obtundation, absent menace response, proprioceptive ataxia, and proprioceptive deficits. Note the absence of the cerebral hemispheres with a small cortical rim and preserved hippocampal structures and the bilateral severe enlargement of the lateral ventricles, suggesting hydranencephaly.

**Figure 3:**
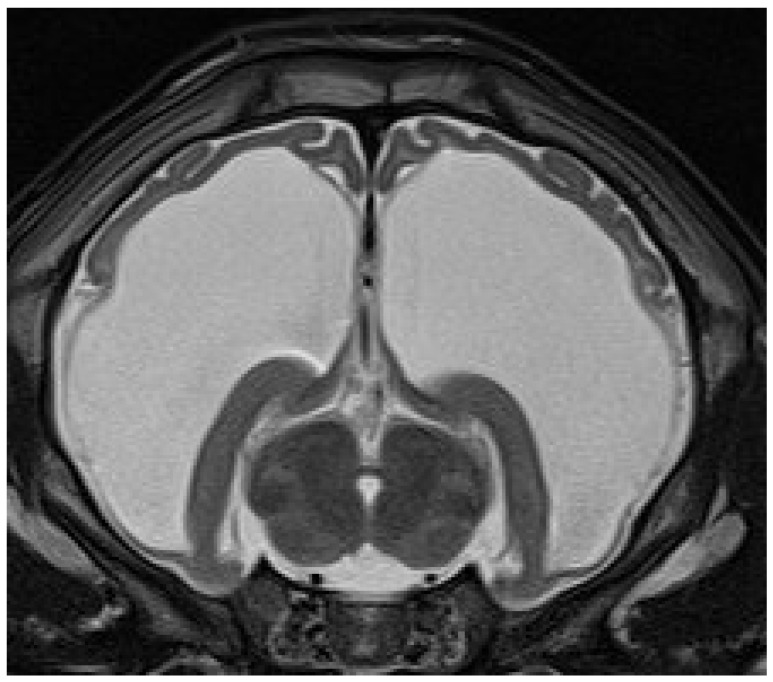
Transverse T2-weighted MRI of a brain at the level of the thalamus and hippocampus of a 28-day-old male calf with obtundation, absent menace response, proprioceptive ataxia, and proprioceptive deficits. The cerebral hemispheres are severely reduced and residual gyrated cerebral tissue is present. The remaining ventricular cavity is markedly enlarged and filled with cerebrospinal fluid. These findings are suggestive of severe internal hydrocephalus.

Eleven calves underwent MRI. Imaging identified different stages of cortical parenchymal loss affecting both white and gray matter throughout the hemispheres in all 11 calves (Table 1). The cerebral hemispheres were reduced to a thin rim of tissue in six cases. The remaining cavity was filled with cerebrospinal fluid by a secondary enlargement of the lateral ventricles (Figure 2). These findings are consistent with hydranencephaly. In three cases, there was a severe enlargement of the ventricular dimensions and residual gyrated cerebral tissue was visible, consistent with severe internal hydrocephalus (Figure 3). Two calves showed no or only mild ventricular enlargement with normal gyration of the cerebral tissue. In these two cases, a reduced contrast between white and gray matter was noticeable and focal hyperintense areas in the white matter were visible. Impaired cerebral myelination or focal leukomalacia were suspected.

### 4.7. Postmortem Results

**Figure 4:**
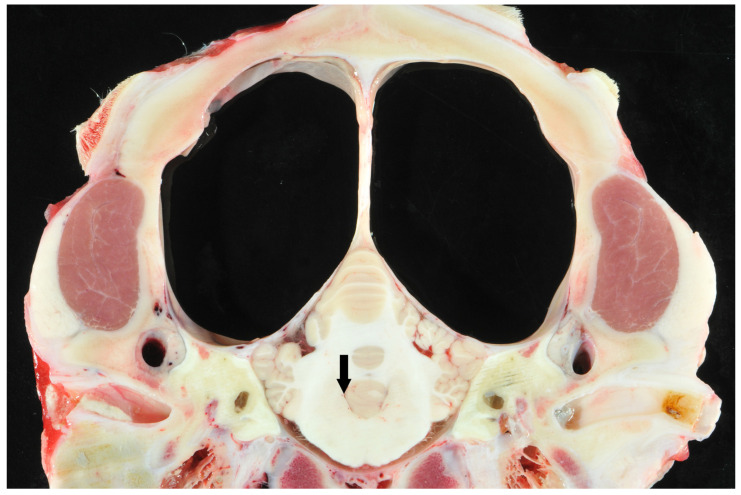
Transverse section of the scull of a calf at the level of the cerebellar vermis and pyramid with suspected hydranencephaly. Note the dilated ventricles and the surrounding small pial rim without visible cortical structures (large clear spaces) and normal cerebellum. The fourth ventricle was not dilated (arrow).

**Figure 5:**
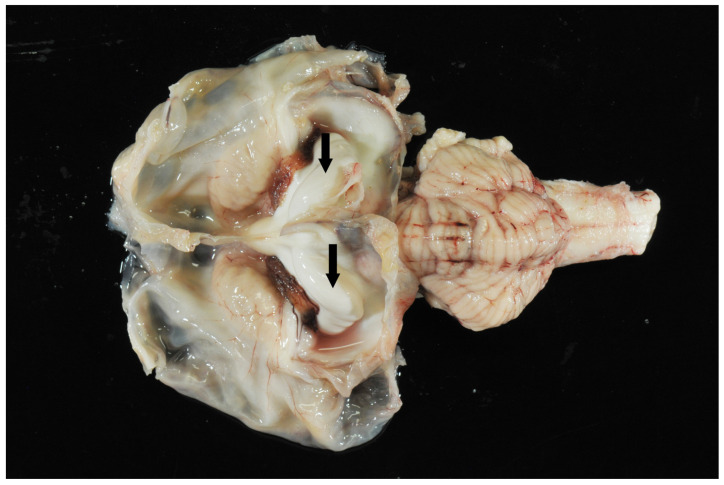
Brain. Hydranencephaly, dilated ventricles, and hippocampus (arrows). Cerebellum and brain stem appear unremarkable.

**Figure 6:**
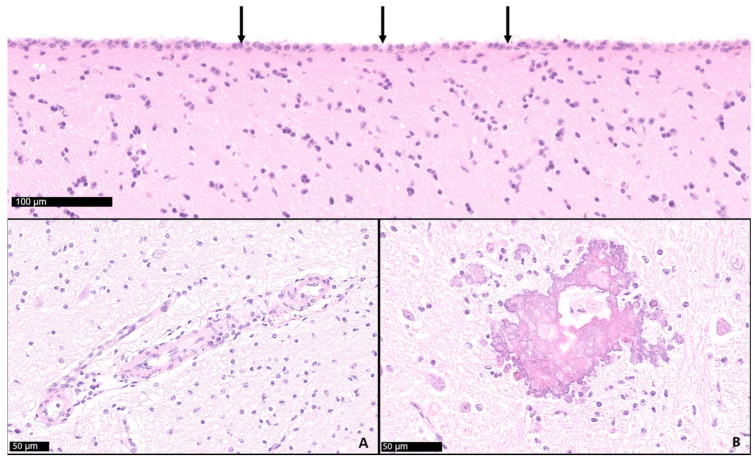
Cerebrum (frontal lobe). The cerebrospinal fluid-filled sacs are lined by ependyma (arrows). Diffuse mild gliosis, mild mononuclear perivascular infiltration (inset **A**), and dystrophic mineralization (inset **B**) observed in cases with hydrocephalus.

While 1 calf only showed mild meningeal edema, 12 out of 13 calves exhibited central nervous system changes, ranging from moderate to severe ventricular dilation (Table 1). Severe hydrocephalus was characterized by bilateral dilation of the lateral ventricles with severe loss of cerebral gray and white matter. In seven cases, cortical parenchyma could not be discerned macroscopically, a finding that is consistent with hydranencephaly (Figure 4). The hippocampus (Figure 5) and spinal cord were not affected in any case. Histopathological examination confirmed the incidence of hydrocephalus in some cases, maintaining at least a thin layer of atrophic cortical white and gray matter and a layer of ependyma lining the ventricles in all animals macroscopically diagnosed with hydrocephalus. Histologically, the brainstem, hippocampus and cerebellum appeared normal in all 13 cases. Periventricular white matter showed foci of mineralization, mild perivascular mononuclear infiltration, and gliosis in one calf with moderate ventricular dilation (Figure 6). Minor concomitant postmortem findings included ulcerative stomatitis (n = 6), suppurative bronchopneumonia (n = 5), and follicular hyperplasia in the spleen (n = 4).

### 4.8. Age Range of Fetus and Presumed Period of Infection of the Dam

**Figure 7:**
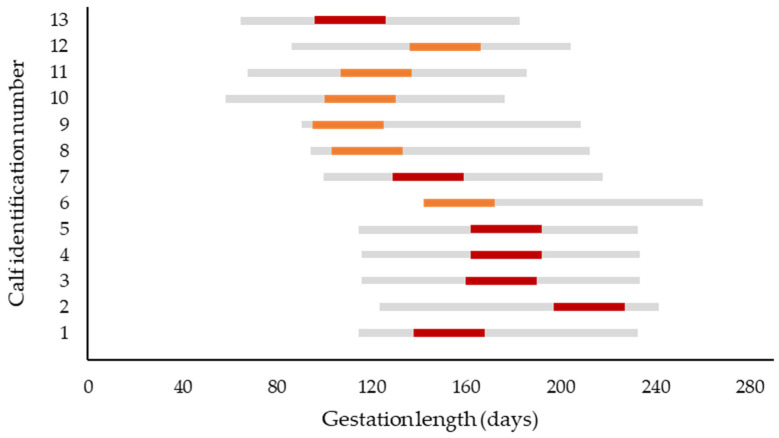
Estimated gestation period at infection of the dam (red bars represent cows from cow–calf operations; orange bars represent dairy cows). The gray bar represents the period of most bluetongue virus serotype 3 infections in Germany (between the 1st of July and 31st of October). The dates of presumed infection of the dams are derived from health records of the farm when available, or from data of the Animal Disease Information System that records positive laboratory results of this notifiable disease by local region [16]. In case the breeding date was unknown (cow–calf operation), the breeding date was calculated as date of calving minus 282 d. To account for uncertainties concerning the actual date of infection, the most probable date ± 15 d is displayed (red and orange bars).

The estimated fetal age at the time of intrauterine infection for each calf is presented in Figure 7. The estimated median gestation length at infection of the dam was 152 d (range: 95 to 227 d of gestation). Differentiating between calves from cow–calf operations and dairy calves, the estimated median gestation length at infection of the dam was 175 d (range: 96 to 227 d) and 122 d (range: 95 to 172 d), respectively.

## 5. Discussion

The present report summarizes results of the diagnostic workup of calves with cerebral malformations that occurred following BTV-3 infection of the dams during pregnancy.

The clinical presentation of affected newborn calves with symptoms present since birth, and which were unresponsive to treatment, show remarkable similarities to the presentation of cases observed during the BTV-8 outbreak in Europe between 2006 and 2008 [23,24,25]. Affected calves showed teratogenic cerebral malformations presumably caused by transplacental infection of fetuses in calves and lambs with BTV-8 and were often characterized as “dummy calves” [12,26,27]. Historically, transplacental transmission of BTV was considered to be a sporadic event, predominantly associated with infection with laboratory-adapted strains of the virus contained in modified live vaccines [28,29]. Field strains of BTV in contrast were considered incapable of transplacental infection of a fetus [9].

In the present case series, the results of the antemortem imaging as well as the postmortem examination consistently revealed marked distention of the lateral cerebral ventricles with concomitant pronounced hypo- or aplasia of cortical parenchyma of the cerebrum. These morphologic findings are consistent with either hydrocephalus or hydranencephaly. Although both conditions are considered to have distinct pathogeneses, the purely morphologic distinction in vivo and postmortem has proved to be challenging [12,13,14]. Whereas hydranencephaly is defined as complete or near complete absence of cerebral hemispheres, with the thalamus, brainstem and basal ganglia being preserved, hydrocephaly is described as an increase in the volume of cerebrospinal fluid in the ventricles, causing ventricular swelling and widespread damage to neuronal structures [13]. In the present case series, detailed pathohistological examination showed that in all cases, the fluid-filled cavities were lined by ependyma; however, an obstruction of the foramen Monroi or foramen Luschka could not be detected. Therefore, this malformation can be considered as hydrocephalus internus ex vacuo. Lesions in the brain of cattle infected with BTV-8 were denominated as hydranencephaly in earlier reports [12,13,14]. Histological evidence to justify the use of the terminus hydranencephaly instead of severe hydrocephalus internus has not been sufficiently expounded.

Lesions of the skin on the muzzle and the oral mucosa observed in several animals might also be associated with BTV infection in affected animals. Similar lesions are commonly observed in ruminants affected by BTV infection [30].

Calves were between 4 and 70 days of age at admission, and per inclusion criteria were required to be positive in the BTV-PCR, indicating that viremia persisted for up to 70 days in calves presumably infected in utero. Several calves were tested repeatedly for BTV-antigen between the first presentation and euthanasia, and in two of these cases, the first sample yielded a positive result, while a second sample was negative.

Viremia persisting for weeks after birth was reported in calves born from dams infected with BTV-8 [12,26]. Bluetongue virus is strongly associated with blood cells during viremia, with the strongest associations with platelets and erythrocytes. Prolonged viremia is a hallmark of BTV infection in ruminants and was explained with the corresponding lifespan of bovine erythrocytes [27]. In healthy calves, the lifespan of erythrocytes has been estimated to be over 100 days [31]. Several studies have found that infectious virus is detectable for a considerably shorter period of up to 9 weeks compared with viral RNA that can be retrieved for up to 140 d [32]. Thus, a positive PCR for BTV RNA does not necessarily imply that the infected animal is infectious to *Culicoides* for the entire period for which RNA can be identified [27]. The hematologic analysis of the calves included in this case series did not reveal any indication for red blood cell abnormalities such as erythropenia or microcytosis, suggesting that although red blood cells were reported to carry viral RNA, this does neither appear to alter cell function nor erythrocyte morphology in a clinically relevant manner.

Early work on BTV and its effect on the fetus led to the hypothesis that transplacental infection may lead to persistent viremia and immune tolerance to the infecting agent [33]. Later work demonstrated that calves do produce antibodies after intrauterine infection and thus are able to mount an immune response whilst the virus is circulating [34,35]. Remarkably, De Clerq et al. [26] reported three calves that were positive for BTV-8 RNA but negative for BTV-8 antibodies in the virus-neutralizing test [26]. The authors concluded that these calves must have been immunotolerant against BTV-8. Repeated testing confirmed that viremia of newborn calves infected in utero declined over the first few weeks of life but could persist for up to 5 months [12,36]. There is currently no indication for the possible occurrence of a persistent (lifelong) viremia after intrauterine BTV infection [37].

In five calves, an increased proportion of platelets was observed. Infection of endothelial cells with BTV may lead to extensive vascular damage with fluid leakage and bleeding associated with thrombocytopenia in the early course of the disease [9]. McColl and Gould observed the nadir platelet concentration in sheep 9 to 13 d post-infection with BTV, but found increasing platelet concentrations afterwards that were greater than the baseline concentrations [38]. The results of clinical and blood biochemical examination indicate that failure of transfer of passive immunity with moderately to markedly decreased plasma protein concentrations was the most common concomitant condition diagnosed in the studied calves. Inadequate consumption of colostrum is likely attributable to the neurologic deficits in affected animals that resulted in reduced suckling activity. Similarly, emaciation and dehydration diagnosed clinically as well as based on elevated blood urea nitrogen concentrations in several animals are likely to be attributed to inadequate feed consumption. A subset of studied calves showed moderately to markedly elevated GLDH activities, as well as elevated concentrations of total bilirubin, suggesting liver cell injury and altered liver function. It is unclear if liver cell lesions were the result of damage directly caused by BTV-3 infection, or if liver cell injury was secondary to emaciation with ensuing liver lipid accumulation [39]. The blood biochemical and blood gas analysis did not reveal any indication for other specific metabolic disturbance or organ damage consistently occurring in studied calves.

Only calves with a positive PCR result for BTV antigen in their blood which were born to unvaccinated dams with a positive titer for BTV antibodies were included in the present study. These inclusion criteria permit the safe assumption that both calf and dam were exposed to BTV. Extrauterine infection of a calf during early life can, however, not be ruled out with certainty. The presence of neurological symptoms since birth and the negligible incidence of reported cases of BTV-3 infections between November 2024 and February 2025, in combination with marginal vector activity during the winter period, make this scenario highly unlikely.

BTV-related malformations of the brain and viremia at birth or in early life are not inherently linked. Studies investigating the BTV-8 outbreak in northern Europe between 2006 and 2009 reported teratogenic effects on the central nervous system in calves with negative test results for the BTV antigen in their blood [24]. In some of the affected animals, viral RNA was retrieved from other tissues such as the spleen or brain [12]. On the other hand, a number of calves without any clinical impairment were diagnosed positive for RNA of BTV at birth [40,41].

The gestational stage at which the fetus is exposed to virus is presumed to be critical for the occurrence of teratogenic effects. Experimental studies identified the time span between 70 and 130 days of gestation as the period of highest risk for cerebral malformations such as hydranencephaly [10,28,42]. In the present study, an attempt was made to crudely estimate the fetal age at the time of BTV-3 infection of the dam (Figure 5). In contrast to the aforementioned studies, our results suggest that in the present study, several calves with neurologic and histopathologic alterations were infected after the 150th d of gestation.

The major limitation of the present case series is that no direct virus detection was performed in the brain lesions. Although it is likely that the observed cerebral malformations are associated with the BTV-3 epidemic, the relation remains at a level of hypothesis. Other infective agents have also been associated with neurologic alterations such as hydranencephaly and hydrocephalus. The viruses causing teratogenic cerebral malformations are Akabane virus [43], Aino virus [44], Peaton virus [45] and BVD [46] in cattle. Border disease was reported to lead to hydranencephaly in sheep and goats [47]. The German National Animal Disease Information System did not, however, record any outbreaks on a regional or national level of infectious disease that were previously related to malformations of the central nervous system in ruminants. All calves were tested for BVD, but not for Akabane, Aino, or Peaton viruses, as these viruses are endemic in tropical and subtropical regions of Asia and Australia and diagnostic tests are not available on a routine basis [48]. Furthermore, inherited hydrocephalus has been described in various breeds [49]. The coincidence of cerebral malformations in calves following the BTV-3 epidemic in summer 2024 in Europe indicate that an inherited cause or another viral infection seems unlikely.

Especially in cow–calf operations, BTV infections of the dam were unnoticed, and therefore no PCR results of dams were available to determine the exact time period of maternal BTV infection. To estimate the fetal age at infection, we referred to data from the German Animal Disease information system. These data give accurate information on the infection incidence in the region where the farm is located, but not on the farm of origin itself, and less so on the dam of the affected calf. Furthermore, in most cow–calf operations the breeding date had to be estimated (date of birth minus 282 d) as no distinct information on breeding was available. To account for these uncertainties, we have included a period of ±15 d around the most probable date of infection in Figure 7.

## 6. Conclusions

In the present case series, we report the diagnostic workup of 13 calves born between November 2024 and February 2025 with congenital neurologic abnormalities, following an outbreak of BTV-3 in Germany in 2024. The pregnant dams were presumably infected between 95 and 227 d of gestation. All calves showed severe clinical neurological symptoms. The MRI exam revealed moderate to severe cerebral malformations so that euthanasia was deemed necessary due to the poor prognosis for survival. Postmortem examinations confirmed severe forebrain abnormalities.

As per patient history, none of the calves responded to medical treatment, which is unsurprising considering the cerebral malformations described here. Currently, prevention remains the sole effective strategy to avoid the occurrence of the described clinical presentation, presumably resulting from transplacental BTV-3 infection. Per emergency decree, three inactivated vaccines against BTV-3 were authorized in the European Union in June 2024, which have in the meantime received definitive approval by European authorities. Vaccination against BTV-3 is currently considered the most effective approach to protect the animals from clinical disease and to contain the risk of transplacental transmission. Whether transplacental infections and corresponding cerebral malformations can be avoided in practice will have to be evaluated.

## Figures and Tables

**Table 1:** Breed, age at admission, particular results of the different examinations, and estimated fetal age at infection of the 13 calves included in the case series.

	Parameter					
No. of Calf Enrolled	Breed and Sex of the Calves	Age at Admission (d)	Clinical Findings	Imaging	Postmortem Examination	Estimated Fetal Age at Infection (d) ^1^
1	Simmental, male	5	Empty chewing, respiratory symptoms	No MRI performed	Hydranencephaly	138–168
2	Limousin, female	14	Hypothermia	Severe hydrocephalus internus	Severe hydrocephalus internus	197–227
3	Gelbvieh, male	14	Hypothermia, hypoglycemia, mucosal lesions, diarrhea, dehydration	Hydranencephaly	Hydranencephaly	160–190
4	Aberdeen Angus, male	28	Hyperthermia, tachycardia, respiratory symptoms, diarrhea	Severe hydrocephalus internus	Severe hydrocephalus internus	162–192
5	Aberdeen Angus, female	28	Hyperthermia, respiratory symptoms, diarrhea	Severe hydrocephalus internus	Severe hydrocephalus internus	162–192
6	Holstein Friesian, female	70	Tachycardia	Hydranencephaly	Hydranencephaly	142–172
7	Rotvieh, female	17	Respiratory symptoms	Impaired cerebral myelination and focal leukomalacia	Mild meningeal edema	129–159
8	Holstein Friesian, male	24	Respiratory symptoms, tachycardia, mucosal lesions	Hydranencephaly	Hydranencephaly	103–133
9	Holstein Friesian, female	16	Hyperthermia, respiratory symptoms, mucosal lesions	Hydranencephaly	Hydranencephaly	95–125
10	Holstein Friesian, male	3	Respiratory symptoms, tachycardia, subluxation of left glenohumeral joint, dehydration	No MRI performed	Moderate hydrocephalus internus	100–130
11	Holstein Friesian, male	10	Hyperthermia, mucosal lesions, tachycardia, diarrhea, dehydration	Hydranencephaly	Hydranencephaly	107–137
12	Holstein Friesian, male	37	Respiratory symptoms, tachycardia	Ventricular enlargement, Impaired cerebral myelination and focal leukomalacia	Moderate ventricular dilation, multiple glial nodules, focal edema, moderate perivascular lymphohistiocytic infiltration	136–166
13	Charolais, male	25	hyperthermia, respiratory symptoms	Hydranencephaly	Hydranencephaly	96–126

^1^ Estimated fetal age at infection is derived from health records of the dam when available, or from data of the Animal Disease Information System, which records positive laboratory results for this notifiable disease by local regions [16].

## Data Availability

All available data are included in this manuscript or the related Supplementary Materials.

## Supplementary Materials

The following supporting information can be downloaded at https://www.mdpi.com/article/10.3390/ani15162359/s1, Table S1: Results of the hematological exam, plasma biochemistry, and blood gas analyses of the 13 calves included in the case series.

## Abbreviations

The following abbreviations are used in this manuscript:

BTV	Bluetongue virus.
BTV-3	Bluetongue virus serotype 3.
BTV-8	Bluetongue virus serotype 8.
BVD	Bovine Virus Diarrhea virus.
PCR	Polymerase chain reaction.
MRI	Magnetic resonance imaging.
MCV	Mean corpuscular volume.
MCHC	Mean corpuscular hemoglobin concentration.
