# Supplementary material for: Cerebral Malformations in Calves Presumed to Be Associated with an Outbreak of Bluetongue Virus Serotype 3 Infection
Source: Animals (Basel). 2025 Aug 11;15(16):2359. doi: 10.3390/ani15162359 (PMC12382794; doi:10.3390/ani15162359)
Supplement: Supplementary file 1 [file animals-15-02359-s001.zip › animals-3717138-supplementary.pdf]

Table S1. Results of the hematological exam, plasma biochemistry, and blood gas analyses of the 13 calves included in the case series.

		Calf identification number												
**Parameter**	**Reference**	**1**	**2**	**3**	**4**	**5**	**6**	**7**	**8**	**9**	**10**	**11**	**12**	**13**
**Complete blood count**														
**White blood cells, $x10^9/L$**	5.60-14.31	20.12	13.85	20.22	9.51	10.01	7.56	6.64	7.14	9.97	5.06	9.41	11.79	8.06
**Neutrophils, $x10^9/L$**	1.19-6.40	13.73	7.39	11.39	1.92	4.34	2.34	2.91	1.85	3.42	2.43	4.64	4.67	2.00
**Lymphocytes, $x10^9/L$**	2.72-8.75	5.41	3.68	7.81	6.76	4.92	4.51	3.32	4.71	6.03	2.06	3.23	6.29	5.43
**Monocytes, $x10^9/L$**	0.07-0.37	0.49	2.06	0.69	0.49	0.42	0.43	0.27	0.33	0.38	0.40	1.11	0.54	0.44
**Eosinophils, $10^9/L$**	0.04-1.52	0.36	0.14	0.04	0.12	0.10	0.02	0.01	0.08	0.04	0.05	0.08	0.05	0.07
**Basophils, $10^9/L$**	0.03-0.23	0.10	0.08	0.11	0.11	0.15	0.10	0.04	0.09	0.09	0.05	0.08	0.11	0.07
**Red Blood cells, $x10^{12}/L$**	5.4-10.6	8.9	6.3	10.1	9.1	8.1	7.4	7.5	6.7	6.9	6.7	6.7	7.9	7.6
**Hemoglobin, mmol/L**	4.7-9.0	7.6	4.9	8.7	6.7	7.2	4.0	6.8	5.0	5.4	6.0	5.1	4.2	5.1
**Hematocrit, %**	21-37	39	24	43	34	35	20	32	26	27	31	26	23	27
**MCV[1],fL**	28.3-50.7	43.6	38.8	43.0	37.7	43.7	27.1	43.1	38.3	39.2	45.4	39.7	29.6	35.0
**Red cell distribution width, %**	16.6-26.7	16.5	18.0	19.1	25.5	19.0	22.8	18.4	19.4	20.4	16.3	19.0	26.6	24.4
**MCH[2], fmol**	0.96-1.25	0.86	0.77	0.86	0.73	0.88	0.54	0.91	0.75	0.78	0.89	0.76	0.54	0.67
**MCHC[3], mmol/L**	21.0-27.3	19.7	20.0	19.9	19.4	20.2	19.9	21.1	19.6	20.0	19.7	19.3	18.3	19.2
**Corpuscular hemogl., mmol/L**	20.3-25.2	19.8	20.7	20.2	20.2	20.6	20.9	21.6	19.7	20.1	19.7	20.5	19.7	20.8
**Platelets, $x10^9/L$**	154-1,022	765	1189	1167	700	633	577	675	1031	1336	424	1511	597	789
**Plasma biochemistry**														
**Total Protein, g/L**	59.2-87.5	60.4	51.2	51.1	54.7	50.1	60.7	39.6	54.0	50.6	58.6	71.6	59.6	51.7
**Albumin, g/L**	29..6-35.9	29.2	23.9	27.7	31.0	27.6	36.0	24.4	32.2	27.2	29.2	31.8	30.5	35.6
**Globulin, g/L**	27.2-49.2	31.2	27.3	23.4	23.7	22.5	24.7	15.2	21.8	23.4	29.4	39.8	29.1	16.1
**Urea, mmol/L**	2.5-5.0	8.2	2.7	19.7	3.5	2.3	5.4	3.2	5	6.3	5.6	5.2	3.2	3.5
**Creatinine, μmol/L**	53-122	76	102	67	159	101	85	48	87	62	81	122	54	117
**Bilirubin total, μmol/L**	<5.3	3.8	4.9	2.6	4.4	10.8	4.1	4.5	6.0	4.0	20.6	6.0	2.6	3.4
**Glutamate dehydrogenase, U/L**	<30	141	10	266	22	9	23	51	9	10	9	10	70	39

**Venous blood gas analyses**														
**Chloride, mmol/L**	93-101	101	90	108	107	105	103	95	95	102	98	102	100	104
**Sodium, mmol/L**	133.3-140.2	140.7	133.9	143.9	143.0	136.8	135.9	129.2	134.2	132.1	137.1	134.5	136.2	137.9
**Potassium, mmol/L**	3.4-4.8	4.6	4.0	3.9	5.1	3.9	4.3	4.5	4.7	4.0	4.3	4.5	4.3	4.3
**Ionized calcium, mmol/L**	1.17-1.37	1.28	1.19	1.19	1.13	1.20	1.25	1.23	1.22	1.25	1.27	1.26	1.27	1.27
**Glukose, mmol/L**	3.9-8.4	6.4	4.3	1.5	7.2	6.3	4.9	3.4	5.3	5.7	6.8	4.6	6.2	4.7
**Lactate, mmol/L**	<2.0	0.72	1.88	0.93	6.48	0.78	0.56	0.83	1.98	0.83	1.81	1.21	1.31	1.31
**Bicarbonate, mmol/L**	26.3-34.1	21.7	34.6	27.2	18.0	26.7	26.3	21.9	32.1	21.8	30.2	26.1	28.9	25.9
**Base-Excess, mmol/L**	2.6-10.8	-3.6	9.0	0.5	-4.7	3.0	1.3	-4.4	6.7	-3.8	5.2	0.5	3.7	0.9
**pH**	7.373-7.466	7.314	7.455	7.377	7.386	7.414	7.357	7.283	7.371	7.305	7.405	7.315	7.375	7.356
**Partial CO_2 pressure, kPa**	5.77-7.82	5.99	6.60	6.13	4.29	5.90	6.55	6.38	7.74	6.09	6.66	7.21	6.83	6.48
**Partial O_2 pressure, kPa**	n.a.	4.68	5.16	3.86	7.10	7.34	6.99	5.98	5.18	7.59	6.18	4.34	4.38	7.58

MCV. Mean corpuscular volume.

MCH. Mean corpuscular haemoglobin.

MCHC. Mean corpuscular haemoglobin concentration.
